# British Hospitals Association and Portsmouth

**Published:** 1921-02-19

**Authors:** 


### British Hospitals Association and Portsmouth.

### HAMPSHIRE REGIONAL COMMITTEE FORMED.

An interesting meeting has been held at Portsmouth to form a regional committee under the British Hospitals Association scheme. Representatives from the Isle of Wight, Chichester, Gosport, and Emsworth met others from Portsmouth. Sir Harold Pink presided, and urged the importance of combination so that the consensus of any body like the hospitals, "attacked" by some Government measure, might be placed before it. Meetings in London cannot be regularly and fully attended by the Association's members, yet the opinion needed is that of the hospitals generally, not that of London hospitals alone. The regions, therefore, proposed should be somewhat similar to those into which the country was divided by the Government for the work of war pensions. These regional committees should be affiliated to the Central Committee, be within measurable distance of each other, and thereby reflect a community of feeling. The Association can then give the considered opinion of its Regional Committees on such important questions as the reintroduction of the Ministry of Health Bill, for example. The voluntary hospitals need a strong association, and decentralisation around a central committee will ensure this. He thinks that the insurance companies will have to listen to such a representative body of opinion.

A precedent on this vexed question exists in the payments made by fire insurance companies towards the maintenance of fire brigades. Besides, the work of his own hospital has much increased since the insurance panel was formed; and as the surplus shown on the insurance funds is something like thirteen millions, some of this balance is due to hospitals for their treatment of insured patients. He therefore moved that a Regional Committee for Hampshire, the Isle of Wight, and part of Sussex be formed.

Mr. Godwin Pratt, of Bournemouth, seconded, and Mr. L. Keyser reminded his audience of the Winchester plan, already described in The Hospital, whereby free treatment is given to those who pay 2d. a week to the hospital. This might be followed elsewhere.

Colonel E. K. Perkins, Southampton, and Mr. H. S. Aylmore, Chichester, spoke in support, and Mr. Pratt emphasised the present, and growing, need of middle-class patients, for whom he suggested a maximum charge of three guineas a week.

The resolution to form a Regional Committee was adopted unanimously. Sir Harold Pink was elected its first chairman, Mr. B. Wagstaff, Royal Portsmouth Hospital, hon. secretary, and Sir Harold Pink, the Committee's representative on the Council of the British Hospitals Association. The post of chairman is to be held on an annual tenure; the secretary for each year is to be secretary of the institution represented by the chairman. Each hospital is to have two representatives on the Regional Committee, but one vote. The meetings will be quarterly, at different towns. The next will be held at Portsmouth.

